# Structural, Thermal, and Storage Stability of *Rapana Thomasiana* Hemocyanin in the Presence of Cholinium-Amino Acid-Based Ionic Liquids

**DOI:** 10.3390/molecules26061714

**Published:** 2021-03-19

**Authors:** Maya Guncheva, Krassimira Idakieva, Svetla Todinova, Denitsa Yancheva, Tsvetelina Paunova-Krasteva, Paula Ossowicz, Ewa Janus

**Affiliations:** 1 Institute of Organic Chemistry with Centre of Phytochemistry, Bulgarian Academy of Sciences, Acad. G. Bonchev Bl. 9, 1113 Sofia, Bulgaria; Krasimira.Idakieva@orgchm.bas.bg (K.I.); Denitsa.Yancheva@orgchm.bas.bg (D.Y.); 2 Institute of Biophysics and Biomedical Engineering, Bulgarian Academy of Sciences, Acad. G. Bonchev Str. 21, 1113 Sofia, Bulgaria; todinova@abv.bg; 3 The Stefan Angeloff Institute of Microbiology, Bulgarian Academy of Sciences, Acad. G. Bonchev Str. Bl. 26, 1113 Sofia, Bulgaria; pauny@abv.bg; 4 Department of Chemical Organic Technology and Polymeric Materials, Szczecin, Faculty of Chemical Technology and Engineering, West Pomeranian University of Technology, Piastów Ave. 42, 71-065 Szczecin, Poland; Paula.Ossowicz@zut.edu.pl (P.O.); ejanus@zut.edu.pl (E.J.)

**Keywords:** ionic liquids, hemocyanin, DSC, FTIR, antibacterial activity, antibiofilm formation, storage stability

## Abstract

Novel biocompatible compounds that stabilize proteins in solution are in demand for biomedical and/or biotechnological applications. Here, we evaluated the effect of six ionic liquids, containing mono- or dicholinium [Chol]_1or2_ cation and anions of charged amino acids such as lysine [Lys], arginine [Arg], aspartic acid [Asp], or glutamic acid [Glu], on the structure, thermal, and storage stability of the *Rapana thomasiana* hemocyanin (RtH). RtH is a protein with huge biomedicinal potential due to its therapeutic, drug carrier, and adjuvant properties. Overall, the ionic liquids (ILs) induce changes in the secondary structure of RtH. However, the structure near the Cu-active site seems unaltered and the oxygen-binding capacity of the protein is preserved. The ILs showed weak antibacterial activity when tested against three Gram-negative and three Gram-positive bacterial strains. On the contrary, [Chol][Arg] and [Chol][Lys] exhibited high anti-biofilm activity against *E. coli* 25213 and *S. aureus* 29213 strains. In addition, the two ILs were able to protect RtH from chemical and microbiological degradation. Maintained or enhanced thermal stability of RtH was observed in the presence of all ILs tested, except for RtH-[Chol]_2_[Glu].

## 1. Introduction

Ionic liquids (ILs) are salts consisting of bulky asymmetric organic cations and organic or inorganic anions. They have melting points below 100 °C; they are not volatile and have lower vapor pressures in comparison to the organic solvents [1]. Their physicochemical properties are easily tunable by appropriate selection or modification of either the cation or the anion, which is the reason for their versatile applications as catalysts, solvents, CO_2_ storage materials, electrolytes, and many others [2,3,4,5,6,7]. In the recent decade, research into the biotechnological and biomedical applications of ILs has expanded. For example, ILs have shown great potential to be applied in the development of novel drug formulations as excipients, based on them can be developed drug formulations with new routes of administration, or they can be involved in the preparation of dual-active drug formulations [8,9,10]. Numerous studies have shown that, when used as additives or co-solvents, some ILs can preserve the structure and stability of proteins and enzymes during their storage and/or isolation. For example, the recovery of lysozyme after extraction with tetraalkylammonium ionic-liquid-based aqueous biphasic systems reaches up to 99% [11]. Surface-active ILs containing cholinium, 1,3-dialkylimmidazolium, tetraalkylammonium, and tetraalkylphosphonium cations and an anion halogen may also facilitate the simultaneous separation of immunoglobulin G and human serum albumin from plasma and serum samples [12]. In addition, aqueous two-phase systems containing ILs based on cholinium, 1,3-dialkylimidazolium, tetraalkylammonium, tetraalkylphosphonium, and guanidinium cations and anions carboxylic acids, amino acids, or biological buffers, prove to be highly efficient for the separation and isolation of serum albumin, myoglobin, hemoglobin, trypsin, papain, ovalbumin, cytochrome C, immunoglobulin Y, horseradish peroxidase, alcohol dehydrogenase, cyclodextrin glycosyltransferase, etc. [13]. Enhanced enzyme activity and/or prolonged stability in reaction mixtures containing 5–20% (*v/v*) ionic liquid additives have also been reported. For example, cellulases from *Halorhabdus utahensis* and *Saccharomyces cerevisiae* showed up to 120% relative activity in media containing 1-ethyl-3-methyl imidazolium diethyl phosphate in comparison to the reaction conducted in buffer only [14]. Enhanced thermal stability and up to 140% relative activity was reported for endoglucanase from *Aspergillus niger* in the presence of tris(2-hydroxyethyl)-methylammonium methylsulfate [14]. High conversion rates (98.5–99%) and relative activity enhanced by 50 to 80% was observed for various alcohol dehydrogenases tested in the presence of up to 50% aqueous solutions of ILs containing 1-butyl-3-methylimidazolium or dialkyl bis(2-hydroxyethyl) ammonium cations and halide, hexafluorophosphate and bis(trifluoromethylsulfonate) anions [14]. Similarly, enhanced enantioselectivity, higher conversion rates, and/or enhanced kinetic thermal stability were reported for numerous aqueous solutions of lipolytic enzymes or hydrophobic organic solvents containing 1,3-dialkyl imidazolium halides, bis(trifluoromethylsulfonates), or hexafluorophosphates [15,16,17,18]. Some ILs based on 1,3-dialkylimidazolium cations can improve the solubility of proteins which are poorly soluble in an aqueous media, even under harsh reaction conditions (high temperature, extreme pH, etc.), such as wool and hair keratins, and plant proteins e.g., zein protein, assist the remodeling/reprocessing of silk and therefore enhance the utilization of these bioresources [14]. Structural refolding of urea-denatured lysozyme was observed in the presence of 1% hydrophobic ammonium-based ILs with trifluromethylsulfonyl imide anion, while tetraethylammonium nitrate and tetrabutylammonium nitrate prevented hen egg-white lysozyme from aggregation [19]. Nώ-alkyl- and Nώ-(hydroxyalkyl)-N-methylimidazolium chlorides with alkyl chain lengths of two to six carbon atoms can act as refolding enhancers of the hen egg-white lysozyme and the anti-oxazolone single-chain antibody fragment [19]. Our recent research has shown that, when added to acidic aqueous media, 1-butyl-3-methylimidazolium acetate, 1-butyl-3-methylimidazolium trifluoroacetate, cholinium L-glutamate, and dicholinium aspartate stabilize the monomeric form of insulin and significantly enhance its thermal stability [20,21].

Hemocyanins (Hcs) are large copper-containing glycoproteins with an oxygen-transporting function that are freely dissolved in the hemolymph of mollusks and arthropods [22,23]. They have complex multimeric structures and, depending on their origin, their molecular weight varies between 3.5 and 13.5 MDa [22,24]. Each subunit is approximately 400 kDa and consists of eight functional units, each possessing a type-3 copper center [24]. Hcs have been the focus of many investigations due to their huge biomedical potential. Numerous studies, including clinical trials, reveal their potency to induce a non-specific immune response against various viral pathogens, small molecules including drugs, hormones, peptides, tumor-specific antigens, etc., and can therefore be used as protein carriers or vaccine adjuvants [23]. Some of the Hcs exhibit a cytotoxic effect on cancer cells and do not affect or have a lower effect on the viability of normal cells [25,26,27,28]. We have found that ILs with cholinium or 1-ethyl-3-methylimidazolium cation and anions polar or hydrophobic amino acids induce changes in the secondary structure of Hcs from *Rapana thomasiana* and *Helix pomatia*. However, the IL-Hcs complexes are characterized by enhancing antiproliferative effects against hormone-dependent (MCF-7) and hormone-independent (MDA-MB-231) cell lines, and, in some cases, an enhanced selectivity has been observed [29,30,31]. The effect of ILs on the stability and structure of Hcs has not been studied in detail. The stability of therapeutic proteins in an aqueous solution is of importance for their pharmaceutical application. Hcs can typically be stored after freeze-drying in the presence of sucrose for 1–2 years, or at +4 °C in solutions containing 0.1% sodium azide for few weeks, which, however, is toxic for living organisms and should be removed before use [32,33]. In view of their pharmaceutical application, novel non-toxic excipients that are able to preserve the structure and activity of proteins and enzymes in a solution are yet on-demand for clinical practice. For example, still most of the insulin formulations in clinical use contain the toxic phenol, m-cresol, or both excipients [34]. Dimethyl sulfoxide or dimethylacetamide are used to reduce the viscosity of immunoglobulin G1. However, they reduce the thermal stability of this therapeutic and its storage stability could be compromised [35]. In addition, the American Food and Drug Administration and the European Medicine Evaluation Agency define aggregation as the key issue underlying multiple deleterious effects for peptide- or protein-based therapeutics, including loss of efficacy, altered pharmacokinetics, reduced stability and product shelf life, and induction of unwanted immunogenicity [36]. Hence, biobased non-toxic ionic liquids and organic salts with the potential to stabilize protein drugs in solution are an attractive alternative to organic compounds. The focus of this study is the effect of six ILs containing one or two cholinium cations and an anion a charged amino acid (Lys, Arg, Asp, Glu) on the secondary structure, thermal and storage stability of Hc from *Rapana thomasiana*, a protein with remarkable immunostimulatory and immunomodulatory activities with potential to be applied as adjuvant and protein carrier. In addition, the potential of the six ILs as bacteriostatic agents were evaluated. Our results provide more insight into the stability of therapeutic proteins in the presence of bio-based ILs. Bio-based ILs can be used as an alternative to toxic excipients to enhance the storage stability of proteins in solution at ambient conditions. Knowledge gained from our study can be useful for designing new, non-toxic ILs, with potency to preserve structural and/or thermal stability of proteins and enzymes utilized in protein crystallization, isolation, or cryopreservation.

## 2. Results and Discussion

### 2.1. Antibacterial and Antifouling Activity of the Cholinium Amino Acids

**Scheme 1:**
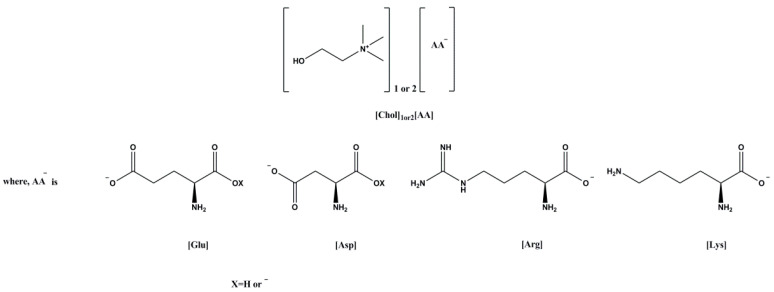
Structures of the tested cholinium-based amino acid ionic liquids [Chol]_1or 2_[AA].

The structures of the six tested cholinium-based amino acids are shown in Scheme 1.

For the antibacterial evaluations, three Gram-negative and three Gram-positive strains) were used, including, E. coli 420, E. coli 25 922, P. aeruginosa PAO1, S. aureus 29213, S. saprophyticus 15305, and B. subtilis 168. The estimated 50% inhibition concentrations (IC_50_) are given in Table 1. Interestingly, [Chol][Glu] and [Chol][Asp] exhibit no effect or even promote the bacterial growth of all tested strains except for S. aureus 29213. Within the series, [Chol][Arg] and [Chol][Lys] seemed to be the most effective antibacterial compounds with an estimated IC_50_ in the millimolar range. The antibacterial activity of these two ILs is in the same range reported in the literature for series of dicationic bromides containing two 1,3-imidazolium cations linked with a short alkyl moiety, and for some piperidinium and guanidinium-based ILs [37,38]. However, [Chol][Arg] and [Chol][Lys] are at least three orders of magnitude less effective against S. aureus, P. aeruginosa, and E. coli than ILs containing imidazolium, piperidinium, morpholinium, and pyrrolidinium cations with long alkyl-chain substituents and bromide anions [37].

We found that S. aureus 29 213 was sensitive to all six tested ILs, while P. aeruginosa PAO1 was resistant to the salts containing anions of the negatively charged amino acid.

We found that despite their weak antibacterial activities, almost all of the six ILs inhibited biofilm formation of the tested microorganisms at a concentration of 1.37 mmol/L, which is lower than the estimated IC_50_ value (Table 2). At this concentration, all compounds showed the highest anti-biofilm formation activity against E. coli 25 922. The reduction of the biomass formation reached up to 95% for S. saprophyticus 15 305 when it was treated with 4.54 mmol/L [Chol][Arg] or [Chol][Lys]. Notably, in an earlier study we found that the six cholinium amino acids have weak to moderate cytotoxic effect on fibroblasts tested at a concentration of 5 mmol/L [21]. In addition, Yazdani et al. have reported that the percentage of biodegradability of monocholinium amino acids is between 75 and 85% tested for 28 days [39]. Our data are scarce for general conclusion, but we can expect that the studied ILs are relatively safe for microorganisms and normal cells, can moderately inhibit biofilm formation, and are readily biodegradable. Thus, they have the potential for use in biotechnological applications.

### 2.2. Effect of the Cholinium Amino Acids on the Conformation of Rapana Thomasiana Hemocyanin

Similarly to almost all hemocyanins, RtH is a blue-colored protein. This is due to the presence of type III copper centers, each functional unit of Hc proteins contains one binuclear copper site (CuAICuBI) [22]. Depending on the oxygen saturation, Hc exists in two main functional forms–oxy-Hc and deoxy-Hc. However, the binuclear copper site of Hcs undergoes a very complex oxidation process, which involves the formation of a series of derivatives with different oxidation states and coordination geometries of the copper ions in the active site [40].

**Figure 1:**
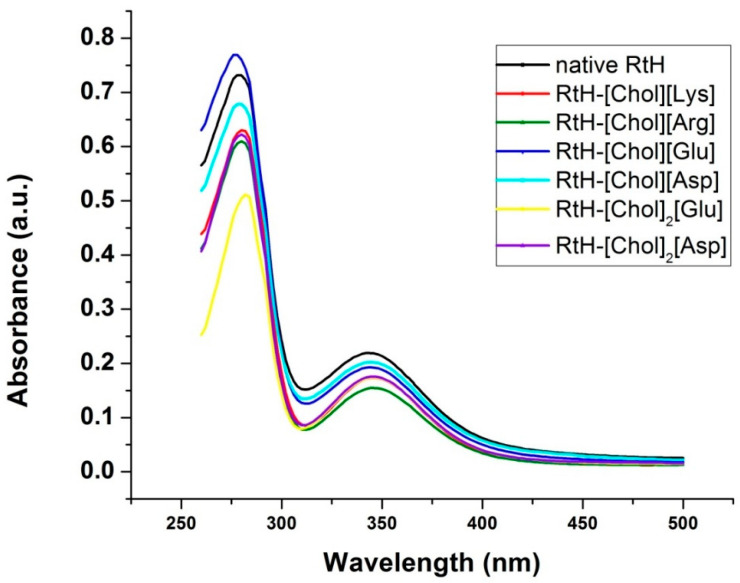
Absorption spectra of *Rapana thomasiana* hemocyanin (RtH) (0.75 mg/mL, 0.08 µM) in 50 mM phosphate buffer (pH 7.2) containing 1.25 mM [Chol]_n_[AA] taken after a 60-min incubation at 20 °C.

Two absorption bands are clearly distinguished in the UV-visible spectrum of RtH (Figure 1).

The observed band at 280 nm is typical for all proteins and is due to the side chains of tryptophan, tyrosine, and phenylalanine residues comprising the molecule. The second band is less intensive and is characteristic of Hc in oxygenated form and is due to the CuI–O_2_^2^—CuI (copper-oxygen complex). The ratio of A345 to A280 as high as 0.25 for RtH is indicative of complete protein oxygenation [29]. Except for the RtH-[Chol][Glu], a decrease in the two absorption bands is observed for all other complexes (Table S1, supplementary). On the other hand, for all complexes, excluding RtH-[Chol]_2_[Glu], the A345/A280 ratio is the same as that estimated for the native protein, which suggests that the geometry of the binding site is preserved, and the oxygen-binding capacity seems unaffected. Interestingly, in the presence of [Chol]_2_[Glu] this ratio is even higher, which is probably due to conformational changes ensuring more efficient electron transfer within the copper-oxygen complex. The effect of [Chol]_2_[Glu] and [Chol][Glu] on the position and intensity of the band at 280 nm in the RtH spectrum is the opposite. While the dicholinium derivative causes a red-shift and a significant decrease (by approx. 40%) in the intensity of this band, the monocholinium-derivative induces a blue-shift of the band at 280 nm and a 10%-increase in its intensity in comparison to the control (native protein only).

**Figure 2:**
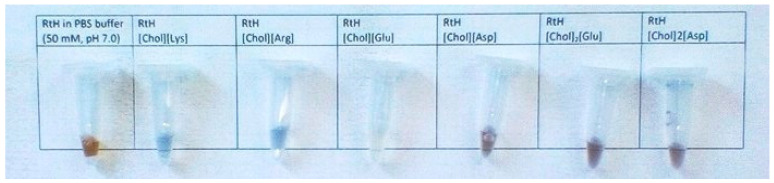
Samples stored at room temperature for 3 months.

No visible spectral changes were observed in the RtH samples containing [Chol][Arg] or [Chol][Lys], even if the solutions were kept for three months at room temperature under non-sterile conditions (Figure 2). It seems that these two ILs protect the protein solution from microbial contamination and chemical changes. We observed browning of the samples of RtH in buffer only and in the presence of [Chol]_2_[Glu], [Chol][Asp], and [Chol]_2_[Asp], which is probably due to a non-enzymatic reaction between RtH carbohydrate moieties and amino groups of the protein and/or ILs. In the presence of [Chol][Glu], the non-enzymatic protein browning seems to be suppressed, but during prolonged storage of RtH at room temperature, its characteristic blue color is lost, and its oxygen-transporting function has probably deteriorated.

**Figure 3:**
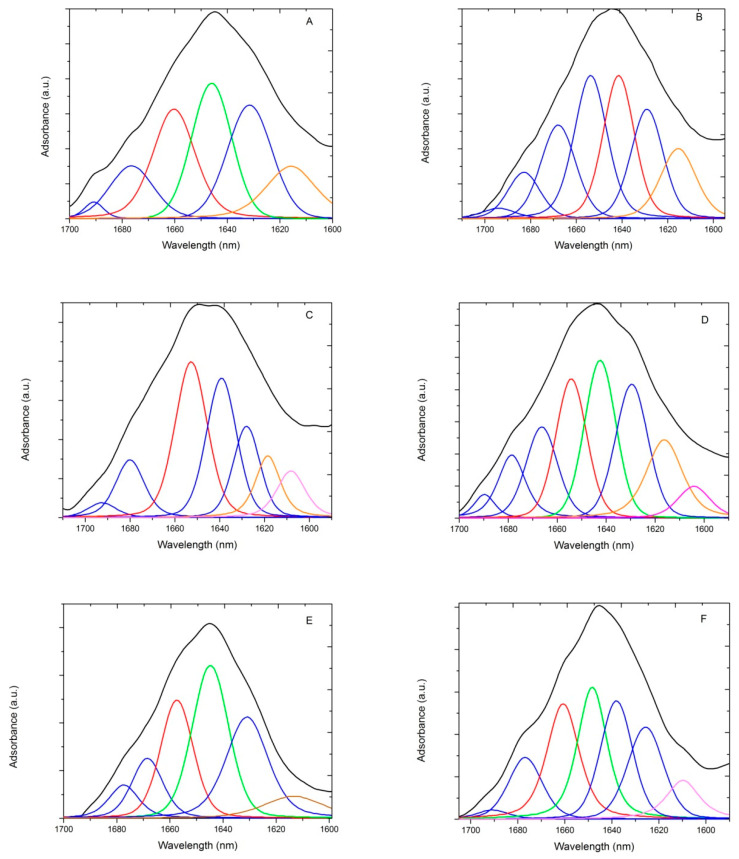

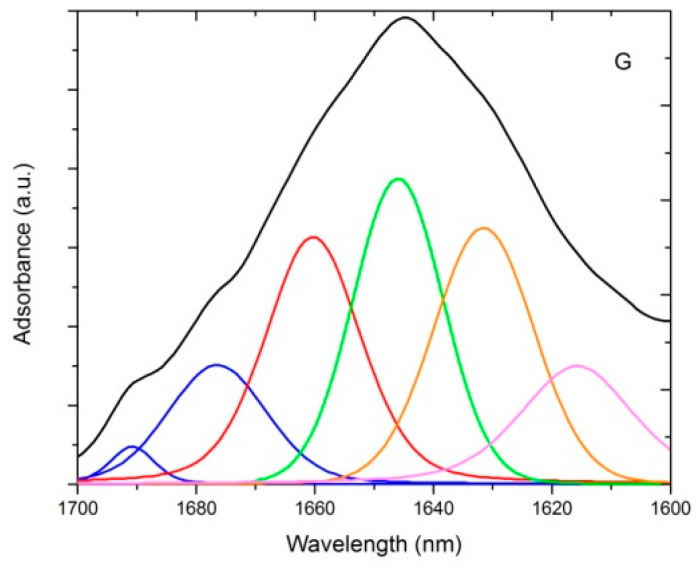
Amide I band with its underlying band subcomponents: α-helices (red), β-structures including β-sheets, β-turns, β-strands, etc. (blue), random coils or unordered structures (green), aggregates (orange), amino acid side chains (mandle) for RtH (30 mg/mL) in 50 mM sodium phosphate buffer (**A**) and in the presence of 0.2 M [Chol][Lys] (**B**), [Chol][Arg] (**C**), [Chol][Glu] (**D**), [Chol][Asp] (**E**), [Chol]_2_[Glu] (**F**), or [Chol]_2_[Asp] (**G**).

By ATR-FTIR spectroscopy, we monitored the changes in RtH secondary structure in presence of the tested ILs. The peak positions in the spectral region 1700–1600 cm^−1^ of the amide I bands are α-helices (1650–1658 cm^−1^), β-sheets (1623–1641 cm^−1^ and 1674–1695 cm^−1^), random coils (1648 cm^−1^), antiparallel β-sheets or β-aggregates (1610–1620 cm^−1^ and 1692–1698 cm^−1^), and side chains of amino acids (1600–1610 cm^−1^). The deconvoluted ATR-FTIR spectra for the native RtH and its complexes with [Chol][AA] are given in Figure 3.

**Figure 4:**
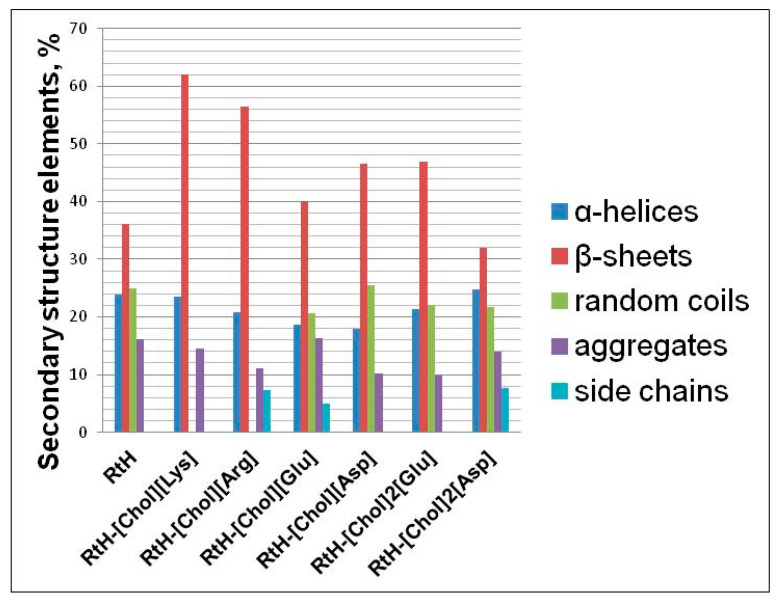
Conformational changes of RtH (30 mg/mL) in the presence of 0.2 M [Chol]_n_[AA] monitored by ATR-FTIR.

The share/portion of each component is expressed as a percentage of the total amide area. The changes in the secondary structure of RtH due to its interactions with the ILs are illustrated in Figure 4 for a comparison.

In the presence of [Chol][Lys] and [Chol][Arg], we observed the most significant increase in the content of β-structures and a complete loss of the unordered structures, while the α-helical structures were preserved. In these two ILs, the structure seems more ordered. Similarly, in a previous study, we found that [Chol][Lys] and [Chol][Arg] induce unfolding of insulin, while the share of the β-sheets increases on behalf of the α-helices [14]. In the presence of [Chol]_2_[Asp], the structure of RtH is preserved and is very close to the structure of the protein in buffer. More open structure and side-chain amino acids exposed to the environment are observed in the spectra of RtH in the presence of [Chol][Arg], mono[Chol][Glu], and [Chol]_2_[Asp].

### 2.3. Effect of Cholinium Amino Acids on the Thermal Stability of Rapana Thomasiana Hemocyanin

**Figure 5:**
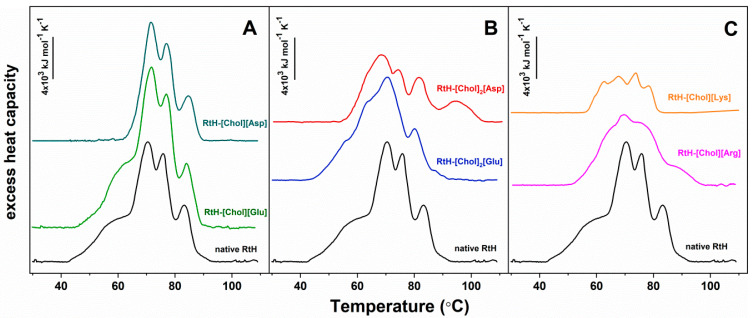
Thermal denaturation profiles of native RtH (3 mg/mL) in sodium phosphate buffer (pH 7.4, 50 mM) in the presence of 0.02 M [Chol]_n_[AA]. Heating rate –1 °C/min. For a comparison the DSC curves are grouped as follows: complexes of RtH with monocholinium salts containing anion of a negatively charged amino acid (**A**), a positively charged amino acid (**B**), and dicholinium salts (**C**).

Differential scanning calorimetry was used to monitor changes in the thermodynamic state of Hc in the presence of ILs. Our data showed that the thermal denaturation of RtH was a complex process and more than one structural domain was observed in the analyzed samples (Figure 5).

The thermodynamic parameters obtained after the experimental deconvolution of thermal unfolding of RtH are given in Table 3. The DSC thermogram of RtH dissolved in sodium phosphate buffer only showed three main endothermic maxima centered at 70.2, 75.8, and 83.3 °C, and one lower temperature shoulder, centered at 60 °C. The result is in agreement with earlier reports where the multiple peak profile of RtH thermal denaturation was attributed to a simultaneous, relatively independent thermal unfolding of several structural domains or subunits. As it has been previously suggested, the sodium phosphate buffer possibly withdraws the calcium and magnesium ions from RtH and induces multi-stage dissociation of the protein [41].

We also observed that the thermal denaturation of all IL-treated RtH samples was a complex process (Figure 5). The differences in their DSC profiles as compared to the non-treated ones, we ascribe to the IL-induced changes in RtH secondary structure, e.g., different degrees of unfolding and/or different rearrangement of the secondary structural elements of the RtH subunits, which were also observed in FTIR spectra. The thermal stability of Hcs has been found to be highly dependent on the presence of Ils. As it can be clearly seen, the smallest effect on Rth conformational stability had [Chol][Glu] and [Chol][Asp]. In the latter, the first broad-temperature transition at about 60 °C was not observed when compared to the non-treated Rth (Figure 4, panel A). [Chol][Lis] and [Chol][Arg] Ils had the strongest effect on Rth temperature stability (Figure 4, panel C). The transitions were not well resolved and had a lower enthalpy compared to the respective peaks of the other cases. We assume that [Chol][Lis] and [Chol][Arg] ILs had the opposite effect on the Rth subunits, and the stabilization of the first and the destabilization of the next two components was the reason for the transitions overlapping. Interestingly, the two dicholinium salts have opposite effects on the thermodynamic stability of RtH. We observed that in the presence of [Chol]_2_[Glu], the four RtH thermal transitions were shifted toward lower temperatures with 4.4, 5.9, 4.7, and 2.7 °C, respectively. Reverse effect, i.e., stabilization of the transitions of RtH in a solution containing [Chol]_2_[Asp] by 9, 5.1, 6.7, and 11.8 °C, was observed. Interestingly, insulin also exhibited an enhanced thermal stability in [Chol]_2_[Asp] [21].

## 3. Materials and Methods

### 3.1. Materials

*Rapana thomasiana* hemocyanin (RtH) was isolated and purified as previously described [22]. The ILs tested in this study- four mono- and two dicholinium salts of charged amino acids i.e., (2-hydroxyethyl)trimethylammonium L-glutamate [Chol][Glu], (2-hydroxyethyl)trimethylammonium L-aspartate [Chol][Asp], (2-hydroxyethyl)trimethylammonium L-argininate [Chol][Arg], (2-hydroxyethyl)trimethylammonium L-lysinate [Chol][Lys], bis(2-hydroxyethyl)trimethylammonium L-glutamate [Chol]_2_[Glu] and bis(2-hydroxyethyl)trimethylammonium L-aspartate [Chol]_2_[Asp], were synthesized and characterized as described in [21].

Bacterial strains *Escherichia coli* 420 and *Bacillus subtilis* 168 were obtained from the Bulgarian national bank for industrial microorganisms and cell culture (NBIMCC). *Pseudomonas aeruginosa* PAO1 was used from the International Reference Panel [42]. *Escherichia coli* 25922, *Staphylococcus aureus* 29213, *Staphylococcus saprofiticus* 15305, and *Bacillus subtilis* 168 were purchased from the American type culture collection (ATCC). Mueller Hinton Broth for microbiology was obtained from Millipore. All solvents were analytically graded.

### 3.2. Antibacterial and Antibiofilm Activity of the Cholinium Amino Acids

Antibacterial and the antibiofilm activity of the series choline amino acids was assessed against three Gram-negative strains (*Escherichia coli* 420, *Escherichia coli* 25922, *Pseudomonas aeruginosa* PAO1) and three Gram-positive strains (*Staphylococcus aureus* 29213, *Staphylococcus saprofiticus* 15305, and *Bacillus subtilis* 168). Activities were estimated in 96-well microplates. At first, bacteria were incubated overnight in Mueller Hinton broth at 37 °C. Then, bacterial cultures from each strain (1 × 10^9^ cells/mL) were diluted 1:100 in the solutions of the tested ionic liquids. Aliquots of 150 µL of these suspensions were pipetted into each well in 6 replicates. Similarly prepared bacterial suspensions in the absence of ionic liquids (buffer only) were used as controls. The plates were wrapped with parafilm to prevent desiccation and were incubated at 37 °C for 24 h without shaking. After the incubation, the absorbance of the plates was measured on an ELISA reader at 620 nm wavelength. For the estimation of biofilm biomass, semi-quantitative crystal violet (CV) assay was applied. The non-adherent bacteria were removed, and the wells were rinsed three times with PBS, stained with 0.1% crystal violet, and incubated at room temperature for 15 min. Then the plates were rinsed several times with sterile distilled water to remove the unabsorbed stain. The samples of Gram-negative bacteria were solubilized with 70% ethanol and these of Gram-positive strains with a dilution of 95% ethanol and acetone (4:1). The absorbance was measured at 570 nm. Control experiments without ILs were performed, and the percentage of inhibition of the biofilm formation was calculated with respect to the controls. All experiments were performed in triplicate. Data were processed by Origin Pro 6.1 software and are expressed as a mean value along with the standard deviation (SD).

### 3.3. UV-Vis Spectroscopy

The UV-vis absorption spectra of Hc and its complexes with [Chol]n[AA] were recorded on a Thermo Fisher Scientific Evolution 300 spectrophotometer (Waltham, MA, USA) in the range of 260–500 nm. Hc (0.75 mg/mL; 0.08 µM) was incubated with 1.25 mM of [Chol]n[AA] both dissolved in 50 mM sodium phosphate buffer for 60 min at 25 °C. To check the stability of the samples, the spectra were recorded at time intervals from 24 h to 168 h. The reference cuvette was filled with sodium phosphate buffer (pH 7.4, 50 mM) and contained the same concentration of the corresponding IL.

### 3.4. Fourier Transformed Infrared Spectroscopy (FTIR)

FTIR spectra of the Hc-IL complexes were recorded on Brucker Tensor 27 spectrometer (Billerica, MA, USA), equipped with a detector of deuterated triglycinesulphate. The spectra were collected in the frequency region of 4000 to 700 cm^−1^ with 128 scannings at a resolution of 2 cm^−1^ by direct deposition of the samples on an attenuated total reflectance element (ATR), a diamond crystal. Complexes were prepared 60 min prior to the measurements by mixing Hc (30 mg/mL) with 0.2 M [Chol]_n_[AA] in 50 mM sodium phosphate buffer containing (pH 7.4). The spectra of the Hcs were referenced to the respective spectra of 50 mM sodium phosphate buffer, pH 7.4, or 0.2 M solution of the corresponding [Chol]_n_[AA] in the same buffer in order to subtract their absorptions. Each experiment was replicated twice, and the data provided corresponds to the average value ±standard deviation. Spectral changes in the Amide I band region (1700–1600 cm^−1^) were analyzed using Opus software version 5.5. At first, in the studied range, the ATR-FTIR spectra were deconvoluted using a bandwidth of 14 cm^−1^, 2.9 resolution enhancement factor, and Lorentzian lineshape. Then, the second derivative spectra were obtained by Savitzky–Golay algorithm based on 17 smoothing points. The components were approximated by Lorentzian/Gaussian functions and the curve-fitting analysis was performed by the Local Least Square algorithm. Calculations of the percentage amounts of the secondary structure elements were performed by computing the ratios of the band areas assigned to the respective substructure. The assignment of the bands was done according to the literature [43].

### 3.5. Differential Scanning Calorimetry (DSC)

DSC scans were carried out with a DASM 4 (Privalov, BioPribor, Moscow, Rusia)—built-in highly sensitive calorimeter with cell volumes of 0.5 mL, a sensitivity >0.017 mJ K^−1^, and a noise level below 0.05 mW. The scan speed was 1 K/min in all experiments. The complexes of 3 mg/mL Hc with 0.02 M of [Chol]_n_[AA] in 50 mM sodium phosphate buffer (pH 7.4) were prepared prior to the measurements. To check the reversibility of the thermal-induced transitions, a reheating scan of the protein solution was performed. As the thermal denaturation was found to be irreversible, the second scan of the denatured sample was used as an instrumental baseline and then subtracted from the original sample curves. The DSC curves were deconvoluted using a successive annealing procedure as previously described [44]. Molecular mass of 9 MDa was used in the calculation of RtH molar concentration.

## 4. Conclusions

The two of the tested ILs, [Chol][Arg] and [Chol][Lys], exhibit potential to be used as excipients in aqueous solutions of *Rapana thomasiana* hemocyanin. Added into the solution, the hemocyanin is protected from microbial contamination and chemical changes, even when stored for more than three months at room temperature. In contrast to the ILs containing anions of negatively charged amino acids, these two ILs do not affect the thermal stability and the oxygen-binding capacity of the protein. Modification of the cation or selection of another cation may improve the antibacterial efficiency of the tested compounds and make the compounds promising for application in the biopharmaceutical application.

## Figures and Tables

**Table 1:** Antibacterial activity of the [Chol]_n_[AA] series.

Bacterial Strain	IC _50_ # (mmol/L)					
	[Chol][Glu]	[Chol][Asp]	[Chol]_2_[Glu]	[Chol]_2_[Asp]	[Chol][Lys]	[Chol][Arg]
Gram-negative						
*E. coli* 420	no effect	no effect	10.27 ± 0.82	7.29 ± 0.20	3.54 ± 0.16	6.14 ± 0.30
*E. coli* 25 922	no effect	no effect	5.73 ± 0.26	34.9 ± 1.75	2.37 ± 0.04	2.9 ± 0.11
*P. aeruginosa* PAO1	no effect	no effect	no effect	promote bacterial growth ~ 10%	6.40 ± 0.13	20.12 ± 0.37
Gram-positive						
*S. aureus* 29 213	5.91 ± 0.17	41.8 ± 1.19	13.8 ± 0.36	25.6 ± 0.61	5.23 ± 0.05	18.75 ± 0.56
*S. saprophyticus* 15 305	no effect	promote bacterial growth ~ 10%	4.36 ± 0.16	12.5 ± 0.28	1.67 ± 0.13	2.39 ± 0.09
*B. subtilis* 168	promote bacterial growth ~12%	promote bacterial growth ~ 7%	promote bacterial growth ~ 5%	14.7 ± 0.49	4.37 ± 0.22	8.48 ± 0.51

# IC_50_: 50% inhibition concentration * Results are means of three different experiments.

**Table 2:** Effect of [Chol][AA] on biofilm formation in the presence of 1.37 mmol/L [Chol]_n_[AA].

Bacterial Strain	Biofilm Formation Inhibition, % #					
	[Chol][Glu]	[Chol][Asp]	[Chol]_2_[Glu]	[Chol]_2_[Asp]	[Chol][Lys]	[Chol][Arg]
Gram-negative						
*E. coli* 420	NA	NA	53.1 ± 2.7	34.1 ± 1.9 47.1 ±2.5 ^(a)^	46.6 ± 2.3 57.1 ±2.1 ^(b)^	57.2 ± 2.5
*E. coli* 25 922	76.3 ± 3.8	86.5 ± 2.5	91.2 ± 3.4	90.2 ± 4.1	89.2 ± 3.6	89.9 ± 3.4
*P. aeruginosa* PAO1	43.5 ± 2.2	38.2 ± 3.1	49.5 ± 2.8	26.8 ± 1.8 62.6± 2.8 ^(b)^	no effect 32.8± 2.0 ^(b)^	7.8 ± 0.5 60.8± 3.1 ^(b)^
Gram-positive						
*S. aureus* 29 213	40.1 ± 4.3	3.6 ± 2.1 29.4 ± 1.6 ^(b)^	23.9 ± 1.7		5.3 ± 0.9 56.6 ± 3.9 ^(b)^	7.8 ± 0.7 60.9 ± 2.5
*S. saprophyticus* 15 305	27.0 ± 3.1 36.8 ± 2.8 ^(a)^	24.8 ± 1.5 66.7 ± 2.6 ^(a)^	48.2 ± 1.8 87.2 ± 3.6 ^(a)^	40.2 ± 2.9 51.7 ± 2.9 ^(b)^	64.5 ± 2.1 94.5 ± 3.4 ^(b)^	60.7 ± 2.9 95.4 ± 3.1 ^(b)^
*B. subtilis* 168	no effect 38.7 ± 2.0 ^(b)^	44.3 ± 1.9	48.5 ± 1.7	54.9 ± 2.5	42.5 ± 2.8	no effect 35.11 ± 1.7 ^(b)^

# Results are means of three different experiments ^(a)^ Tested concentration–2.27 mmol/L ^(b)^ Tested concentration–4.54 mmol/L.

**Table 3:** The thermodynamic parameters for the thermal denaturation of RtH in presence of [Chol]_n_[AA] determined after a successive annealing procedure.

Parameters Sample	T_m1_ (°C)	∆H_1_ (kJ mol^−1^)	T_m2_ (°C)	∆H_2_ (kJ mol^−1^)	T_m3_ (°C)	∆H_3_ (kJ mol^−1^)	T_m4_ (°C)	∆H_4_ (kJ mol^−1^)	∆H_total_ (kJ mol^−1^)
native RtH	60.0	63,901	70.2	59,132	75.8	47,597	83.3	38696	194,637
RtH-[Chol][Glu]	62.0	74,040	70.4	100,553	77.7	46,898	85.0	23,272	244,962
RtH-[Chol][Asp]			71.4	61,384	77.3	45114	86.4	24,055	128,482
RtH-[Chol]_2_[Glu]	55.6	28,715	64.3	46,586	71.1	72,300	80.6	35,821	183,342
RtH-[Chol]_2_[Asp]	69.0	62,104	75.3	21,030	82.5	24,223	95.1	15,435	127,910
RtH-[Chol][Arg]	64.1	29,106	69.6	34,310	75.6	64,704	85.8	17310	136,582
RtH-[Chol][Lys]	62.3	13,980	68.5	16,213	73.6	13,483	78.2	12,113	56,067

## Data Availability

The data presented in this study are available on request from the corresponding author.

## Supplementary Materials

The following are available online, Table S1: Intensity of the absorption bands at 280 and 345 nm and their ratio for the native *Rapana thomasiana* hemocyanin and its complexes with [Chol]_1 or 2_[AA].

## Sample Availability

Samples of the compounds are available from the authors.
