# Supplementary material for: Structural, Thermal, and Storage Stability of Rapana Thomasiana Hemocyanin in the Presence of Cholinium-Amino Acid-Based Ionic Liquids
Source: Molecules. 2021 Mar 19;26(6):1714. doi: 10.3390/molecules26061714 (PMC8003507; doi:10.3390/molecules26061714)
Supplement: Supplementary file 1 [file molecules-26-01714-s001.pdf]

Structural, thermal, and storage stability of *Rapana thomasiana* hemocyanin in the presence of cholinium-amino acid-based ionic liquids

Maya Guncheva[1], [*], Krassimira Idakieva[1], Svetla Todinova[2], Denitsa Yancheva[1], Tsvetelina Paunova-Krasteva[3], Paula Ossowicz[4], Ewa Janus[4]

[1] Institute of Organic Chemistry with Centre of Phytochemistry, Bulgarian Academy of Sciences, Acad. G. Bonchev Bl. 9, 1113 Sofia, Bulgaria; Maya.Guncheva@orgchm.bas.bg (M.G.); Krasimira.Idakieva@orgchm.bas.bg (K.I.); Denitsa.Yancheva@orgchm.bas.bg (D.Y.)

[2] Institute of Biophysics and Biomedical Engineering, Bulgarian Academy of Sciences, Acad. G. Bonchev Str. 21, 1113 Sofia, Bulgaria; todinova@abv.bg (S.T.)

[3] The Stefan Angeloff Institute of Microbiology, Bulgarian Academy of Sciences, Acad. G. Bonchev Str. Bl. 26, 1113 Sofia, Bulgaria; pauny@abv.bg (Ts.P-K.)

[4] West Pomeranian University of Technology, Szczecin, Faculty of Chemical Technology and Engineering, Department of Chemical Organic Technology and Polymeric Materials, Piastów Ave. 42, 71-065 Szczecin, Poland; Paula.Ossowicz@zut.edu.pl (P.O.); ejanus@zut.edu.pl (E.J.)

* Corresponding author: Maya.Guncheva@orgchm.bas.bg (M. G.)

Table S1. Intensity of the absorption bands at 280 and 345 nm and their ratio for the native *Rapana thomasiana* hemocyanin and its complexes with $[Chol]_{1\ or\ 2}[AA]$.

RtH-$[Chol]_{1or2}$ [AA]	A_{345}	A_{280}	A_{345}/A_{280}
native RtH	0.19	0.73	0.26
RtH-[Chol][Lys]	0.16	0.63	0.253
RtH-[Chol][Arg]	0.15	0.61	0.245
RtH-[Chol][Glu]	0.19	0.76	0.25
RtH-[Chol][Asp]	0.17	0.67	0.253
RtH-$[Chol]_2$[Glu]	0.17	0.50	0.34
RtH-$[Chol]_2$[Asp]	0.16	0.62	0.258

RtH (0.75 mg/mL, 0.08 µM) mixed with 1.25 mM [Chol]n[AA] in phosphate buffer (50 mM, pH 7.2); Incubation time: 60 min; T = 20ºC.
